# Does exposure to interparental violence increase women’s risk of intimate partner violence? Evidence from Nigeria demographic and health survey

**DOI:** 10.1186/s12914-018-0143-9

**Published:** 2018-01-11

**Authors:** Bola Lukman Solanke

**Affiliations:** Department of Demography and Social Statistics, Obafemi Awolowo University, Ile-Ife, Nigeria

**Keywords:** Interparental violence, Intimate partner violence, Women, Nigeria, Sexual and reproductive health

## Abstract

**Background:**

Exposure to interparental violence (EIPV) has been identified as a risk factor for intimate partner violence (IPV). However, studies in Nigeria have rarely and specifically examined exposure to interparental violence as a predictor of IPV. The objective of the study was to examine the relationship between exposure to interparental violence and women’s experience of intimate partner violence.

**Methods:**

The 2013 Nigeria Demographic and Health Survey (NDHS) women recode dataset was analysed. The weighted sample size was 19,925 women aged 15–49 years. The outcome variable was women’s experience of at least one type of IPV measured by combining partner physical, sexual and emotional violence experienced by the surveyed women. The main explanatory variable was exposure to interparental violence measured by response to question on whether a woman witnessed her father ever beat her mother. Individual/relationship and community characteristics were selected for statistical control in the study. The multilevel mixed-effect regression was applied in three models using Stata version 12. Model 1 was based solely on interparental violence, while individual/relationship factors were included in Model 2. In Model 3, all research variables were included.

**Results:**

The study revealed that less than one-tenth of the women witnessed interparental violence, and women exposed to interparental violence compared with non exposed women had higher prevalence of all forms of IPV. In Model 1, women exposed to interparental violence were more than five times as likely as non exposed women to experience IPV (OR = 5.356; CI: 3.371–8.509). In Model 2, women exposed to interparental violence were nearly five times as likely as non exposed women to experience IPV (OR = 4.489; CI: 3.047–6.607). In Model 3, women exposed to interparental violence were four times as likely as non exposed women to experience IPV (OR = 4.018; CI: 2.626–6.147).

**Conclusion:**

The study provided additional evidence that exposure to interparental violence increase women’s risk of IPV in Nigeria. Reducing future prevalence of intimate partner violence may require social and behaviour change communication (SBCC) that not only change perception of children who witnessed interparental violence, but also help them to overcome intergenerational effects of interparental aggression.

## Background

Intimate Partner Violence (IPV) refers to any behaviour within a marital union or an intimate relationship that may cause physical, psychological or sexual harm to one or both partners in the union or intimate relationship. Its dominant types include physical violence such as wife-battery, sexual violence such as rape, emotional violence such as intimidation and humiliation, and controlling behaviours such as restricting women’s association with friends and relatives [1]. Across the world, IPV has been widely reported against both men and women [2–7] with both men and women being either the perpetrator or victim of IPV. However, men tend to be more perpetrators of severe forms of IPV [8–12]. A recent global estimate of IPV prevalence reveals that slightly more than one-third of women across the world had experienced at least one type of IPV [13]. In sub Saharan Africa, IPV is not only pervasive, but also widely reported against pregnant women [14]. Research has provided ample evidence of the health and other deleterious effects of IPV. This includes but not limited to homicide [15, 16], suicide attempts [17], poor mental health [18], and several adverse reproductive health outcomes such as pregnancy termination [19], gynaecologic morbidities [20], and posttraumatic stress disorders [21]. IPV is thus a public health and human right crisis across the world [22].

The causes of and risk factors for IPV have been widely situated within the ecological framework [23, 24]. Based on the framework, IPV as well as other forms of gender-based violence is influenced by multiple factors within the social environment. These factors include individual factors (such as age, education, exposure to interparental violence, and acceptance of violence), relationship factors (such as multiple sexual partners, and partner education), community factors (such as gender norms), and societal factors (such as poverty) [1]. Several studies across the world have provided supportive evidence that IPV results from the interplay of factors that cut across several levels of influence in the society [25–30]. In Nigeria, numerous studies have examined the prevalence and correlates of IPV. On one hand are the studies that examined IPV as a predictor of specific health outcome such as contraceptive use, maternal healthcare use and pregnancy termination [31–33], while on the other hand are the studies that predicted IPV based on contextual characteristics of individual, partners and communities [34–36].

However, studies in Nigeria [34–42] have rarely and specifically examined exposure to interparental violence as a predictor of IPV in the country though exposure to interparental violence has been identified as a risk factor for IPV [1]. In other climes, exposures to interparental violence have been adequately linked to IPV in a number of prospective studies [43–48]. These studies not only confirmed that exposure to interparental violence elevates the risk of IPV; they also identified a number of behavioural factors that mediates the relationship between interparental violence and IPV. The implication of the association between exposures to interparental violence and IPV is often overlooked in the discourse on the prevalence of IPV in Nigeria. In one of the few Nigerian studies that investigated exposure to interparental violence [49], the findings could not be generalised to the whole country because the sample was not nationally representative. In another Nigerian study [50] that analysed a nationally representative sample, the focus was not strictly on women’s likelihood of experiencing intimate partner violence, but on whether women who witnessed interparental violence had higher likelihood of tolerant attitudes towards intimate partner violence. Thus, there is need to expand knowledge of the association between interparental violence and women’s experience of IPV in Nigeria. This is crucial for initiatives that seek to reduce IPV in Nigeria given that children who witnessed interparental violence are often a neglected group in such initiatives in the country. This study attempts to fill this knowledge gap by raising the question: does exposure to interparental violence increase women’s risk of intimate partner violence in Nigeria?

Bandura’s social learning theory [51] and the theory of intergenerational transmission of violence provides the theoretical perspective of the study. Both theories provide explanatory mechanism for why individuals observe the way others behave in the society and also attempt to behave in the same way to confirm their acquisition of the particular behaviour. In particular, the theory of intergenerational transmission of violence asserts that children who witnessed interparental violence are more likely to experience IPV later in life either as victim or perpetrator. Though, few studies have found weak empirical evidence for the assertion [52, 53], but large numbers of studies across the world have provided research evidence to support the theoretical position that witnessing interparental aggression may influence experiencing partner violence either as a perpetrator or as a victim later in life [54–60]. The objective of the study was therefore to examine the relationship between exposure to interparental violence and women’s experience of intimate partner violence in Nigeria. This was with the view to providing not only additional information about an underlying cause of intimate partner violence in the country, but also providing information that could help improve future level of women’s sexual and reproductive health in Nigeria. The study was guided by the hypothesis that exposure to interparental violence has significant effect on women’s risk of intimate partner violence.

The study was conducted in Nigeria, the most populous country in Africa [61]. Intimate partner violence is one of the cultural practices that continually affect women’s health and socio-economic rights in Nigeria [62]. Though, the constitution of Nigeria prohibits discrimination against women [63], and the county is a signatory to United Nation’s Convention on the Elimination of all Forms of Discrimination against Women (CEDAW) and other major international women’s health advocacy groups, the country still lags behind many African countries in terms of gender equality and women’s health [64]. Until recently, public efforts to reduce the prevalence of IPV and other forms of domestic violence in the country have been greatly hindered by lack of a national law to criminalise major forms of gender-based violence, though a national gender policy exist in the country to promote women’s health and status [63]. The policy outlined a number of Behaviour Change Communication programmes that aim to mainstream gender into all aspect of the national life, but the policy is now been reviewed [65].

However, several gender activists, women-centred organisations and civil society groups in the country under the auspices of the National Coalition on Affirmative Action (NCAA) have sustained agitation for legislations to protect human and women’s rights in the country. A number of States in the federation such as Lagos, Ekiti, Edo, Ebonyi, Jigawa and Cross River States have enacted specific laws to prohibit several types of gender based violence such as wife battery, harmful widowhood practices and female genital mutilation [65]. The first national legislation against violence was enacted in 2015. The law seeks to prohibits all acts of violence against persons whether male or female, it offers wide range protection for men and women, sought remedies for victims of domestic violence, prescribes punishment for perpetrators of violence against persons in the country, and set up a government agency (National Agency for the Prohibition of Trafficking in Persons and Other related matters) to implement its provisions in collaboration with faith-based organisations in the country [66]. However, the law did not prescribe any form of counselling, treatment or action for children who witnessed interparental aggression. This may have implications for intergenerational transmission of domestic violence in the country.

## Methods

### Data source and sample design

The study analysed women’s data from the 2013 Nigeria Demographic and Health Survey (NDHS). The 2013 NDHS is a nationally representative sample in which samples were randomly selected using a stratified three-stage cluster design based on 904 clusters. A total of 38,948 women were covered in the survey, but only one woman per household was selected for the domestic violence module. Intimate partner violence in the survey was measured based on a modified version of the Conflict Tactic Scale. Competent field staffs were recruited by the NDHS technical team. Irrespective of educational attainments, field staffs were trained in a four-week training course based on standard DHS training procedures. Interviewers administering the domestic violence module were instructed not to commence with questions on intimate partner violence until privacy was ensured. Full details of the 2013 NDHS design have been published [67]. Women who were not included in the domestic violence module and women who were not currently married were excluded in the current study. A weighted sample size of 19,925 women was analysed in the study. The data were formally requested from MEASURE DHS, an organisation under the auspices of ICF International, which provide survey assistance to countries particularly developing countries in the collection of wide spectrum population and health data [68]. Authorisation to access and analyse the data were granted by MEASURE DHS with understanding that respondents remain anonymous. The data analysed in the study has not been linked to any individual or household. The study findings are thus, not expected to be injurious to any individual or household.

### Outcome variable

The outcome variable in the study was intimate partner violence. The 2013 NDHS measured three types of intimate partner violence, namely physical, sexual, and emotional violence by asking ever married women whether their current or former male partner ever: said or did something to humiliate them before other people, and whether he ever threatened to harm them or someone close to them. These questions were used to derive partner emotional violence. Ever married women were further asked if their current or former male partners ever: pushed, shook or threw something, slapped, punched, attempted to choked, or threatened or attacked them with knife or other weapons. They were further asked whether the male partner ever twisted their arm or pulled their hair, and if he ever kicked, dragged or beat them up. These questions were used to derive partner physical violence. Partner sexual violence was based on women’s response to whether the male partner ever physically forced them to have unwanted intercourse or perform other unwanted sexual acts. Women who answered in the affirmative were then asked to provide information on the frequency of any of the acts in the last 12 months preceding the survey [67]. In the current study, the set of questions for each type of IPV were combined and dichotomised to reflect whether a woman has ever experienced or never experience the specific IPV. Thereafter, the three types of IPV were combined to reflect whether a woman has ever or never experienced at least one type of IPV. The essence of combining the three types of IPV was to improve statistical precision regarding association between exposures to interparental violence and IPV given that the proportion of women who experienced specific type of IPV particularly sexual violence were smaller compared with women who experienced at least one type of IPV. Hence, analyses in the study focus more on women who ever experienced at least one type of IPV.

### Explanatory and control variables

The main explanatory variable was interparental violence. This was measured by women’s response to whether she witness her father ever beat her mother. Those who reported witnessing interparental aggression were grouped as ‘exposed’ to interparental violence, while those who reported otherwise were grouped as ‘not exposed’ to interparental violence. In addition to interparental violence, few individual/relationship and community characteristics were included in the analysis for statistical control. The variables which include, maternal age, education, attitudes to wife beating, employment status, male dominance in the family, partner education, partner alcohol drink, community poverty level, geographic region and place of residence, were selected for inclusion because a number of previous studies have revealed their associations with IPV [27, 34–36, 50, 69].

Maternal age was categorised into three groups of 15–24, 25–34 and 35–49 years. Maternal and partner education were also categorised into three groups with no formal education and primary education combined as ‘low’, while secondary and tertiary education were grouped as ‘moderate’ and ‘high’ educational levels respectively. Women’s employment status was divided into ‘working’ and ‘not working’. Attitudes towards wife beating was based on responses to whether women think a male partner is justified in beating his spouse given certain circumstances such as: if wife goes out without permission; wife neglects children; wife argues with husband; wife refuses to have sex with husband; and wife burns food. Women who think the husband is justified in beating the wife given all listed circumstances were grouped as ‘violence justified’ while women who thinks the husband is not justified on at least one of the circumstance were grouped as ‘violence not justified’.

Male dominance in the family was based on who had final say on three household decision-making, namely, final say on women’s health issue, final say on large household purchases, and final say on visits to friends/relatives. Households in which women had sole or joint say on at least one of the decisions were grouped as ‘not male dominated’ while others were grouped as ‘male dominated’. Partner alcoholic drink was grouped into ‘partner drinks’ and otherwise. Community poverty level was derived from household wealth quintile. This was done by first obtaining the proportion of women in the poorest wealth category and then aggregating the proportion at the cluster level. The proportion was then divided into three categories of low, medium and high.

### Data analysis

Data analysis was performed at three levels in the study using Stata 12. At the univariate level, percentages and frequency distribution were used to describe sample characteristics and prevalence of intimate partner violence. At the bivariate level, simple cross tabulation was carried out to obtain proportion of women experiencing at least one type of IPV. The unadjusted binary logit regression was then performed to examine association between the outcome and other research variables. The nature of the regression coefficient (positive or negative) indicates the direction of the association. A Variance Inflation Factor (VIF) was performed to detect collinearity among the explanatory variables. Usually, a mean VIF of more than 5 suggest serious collinearity problem [70]. The mean VIF of 2.36 obtained in the study confirmed the absence of collinearity that could distort the relationship between the outcome and other variables of study. At the multivariate level, the multilevel mixed-effect logistic regression was applied to account for the hierarchical nature of the data, and as well explain IPV by selected factors operating at the individual/relationship and community levels as prescribed by the ecological theory. Similar analytical technique has been used in previous studies in Nigeria [36, 50].

The multilevel model was fitted by the *xtmelogit* command [71] in three models in addition to the empty model. Model 1 was based solely on interparental violence, while individual/relationship factors were included in Model 2. The full model included all individual/relationship and community characteristics. The effects of the multilevel model were measured using odds ratios of binary logistic regression for the fixed effects and the Intra-Class Correlation (ICC) for the random effects of the model. However, the ICC was calculated manually as: $$ \rho =\frac{\sigma_u^2}{\sigma_u^2+\frac{\pi^2}{3}} $$ [72], where $$ {\sigma}_u^2 $$ is the variance at the community level and $$ \raisebox{1ex}{${\pi}^2$}\!\left/ \!\raisebox{-1ex}{$3$}\right. $$ is equal to 3.29. The goodness-of-fit of the multilevel model was examined through the Log-likelihood and the Akaike’s Information Criterion (AIC). Both the log-likelihood and AIC were expected to reduce in values as more variables are been added to the model.

## Results

Table 1 presents respondents’ profile. Nearly a quarter of the respondents are in the younger age group of 15–24 years, while more than one-third of respondents are in the two higher age categories. Slightly more than two-thirds of respondents had low level of educational attainment, while less than one-tenth of respondents had high educational attainment. The majority of respondents did not think male partners are justified under any circumstances to beat their wives; however, more than one-third of the women think the male partner is justified in beating his wife under certain circumstances. The majority of respondents were employed as at the time of the survey. Household decision-making in most of the respondents’ households was male dominated. More than half of respondents’ male partners had low level of educational attainment. However, slightly higher proportions of the partners had either moderate or high educational attainment compared with the respondents. The majority of respondents’ male partners consume alcohol. The majority of the respondents live in communities with low proportion of women in the poorest wealth category.

**Table 1:** Respondents Profile, Nigeria, 2013

Characteristic	Frequency	Percentage
Maternal age		
15–24 years	4759	23.9
25–34 years	7631	38.3
35–49 years	7535	37.8
Maternal education		
Low	13,382	67.2
Moderate	5067	25.4
High	1476	7.4
Attitudes towards wife beating		
Violence not justified	12,595	63.2
Violence justified	7330	36.8
Employment status		
Not working	6121	30.7
Working	13,804	69.3
Male dominance in family		
Not male dominated	6380	32.0
Male dominated	13,545	68.0
Partner’s education		
Low	11,623	58.3
Moderate	5577	28.0
High	2725	13.7
Partner drinks alcohol		
Does not drink	16,425	82.4
Drinks	3500	17.6
Place of residence		
Urban	7279	36.5
Rural	12,645	63.5
Total	19,925	100.0
Proportion poorest in community		
Low	9899	49.7
Medium	2677	13.4
High	7349	36.9
Geographical region		
North-central	2753	13.8
North-east	3290	16.5
North-west	7261	36.4
South-east	1666	8.4
South-south	1950	9.8
South-west	3005	15.1
Interparental violence		
Not exposed	18,311	91.9
Exposed	1613	8.1
Partner physical violence		
Never experienced	18,036	90.5
Ever experienced	1889	9.5
Partner sexual violence		
Never experienced	19,186	96.3
Ever experienced	739	3.7
Partner emotional violence		
Never experienced	16,795	84.3
Ever experienced	3130	15.7
At least one type of partner violence		
Never experienced	16,021	80.4
Ever experienced	3904	19.6
Total	19,925	100.0

Source: Authors analysis based on 2013 NDHS

The majority of respondents are rural dwellers; however, slightly more than one-third of the respondents reside in urban areas of the country. Respondents from the Northern region of the country, particularly the North-West geo-political zone are dominant in the sample. The majority of respondents did not witness interparental violence; however, slightly less than one-tenth of the respondents witnessed interparental violence. Nearly one-tenth of respondents had experienced at least one type of partner physical violence, while more than one-tenth of respondents had experienced at least one type of partner emotional violence. Partner sexual violence was the least reported among the women. Overall, one-fifth of the respondents had experienced at least one type of intimate partner violence. As shown in Table 2, women who were exposed to interparental violence compared with non exposed women had higher prevalence of at least one type of partner physical violence (25.4% vs. 8.1%), partner sexual violence (10.4% vs. 3.1%), partner emotional violence (40.0% vs. 13.8%), and at least one type of IPV (44.3% vs. 17.4%).

**Table 2:** Percentage distribution of ever experienced of IPV by interparental violence

Interparental violence	Partner physical violence Number (%)	Partner sexual violence Number (%)	Partner emotional violence Number (%)	Partner physical violence Number (%)
Not exposed	1478 (8.1)	571 (3.1)	2534 (13.8)	3190 (17.4)
Exposed	410 (25.4)	169 (10.4)	596 (37.0)	714 (44.3)
Total	1889 (9.5)	739 (3.7)	3130 (15.7)	3904 (19.6)

Table 3 presents results of simple cross tabulation and unadjusted binary logistic regression. Exposure to interparental violence and women’s experience of intimate partner violence are positively associated (β = 1.327; CI: 1.176–1.477) with higher prevalence of intimate partner violence among exposed women. Maternal age and women’s experience of intimate partner violence are positively associated across all age categories. Though, prevalence of intimate partner violence increased as women’s age increased from 15 to 24 years to 25–34 years, the prevalence of IPV decline at advanced reproductive age category of 35–49 years. Maternal educational attainment had mixed relationship with women’s experience of IPV. The relationship was positive at moderate educational attainment (β = 0.511; CI: 0.370–0.652), but negative at high educational attainment (β = −0.025; CI: -0.266-0.216). Attitudes towards wife beating and women’s experience of IPV are positively related (β = 0.597; CI: 0.460–0.734) with higher prevalence of IPV among women who think the male partner is justified beating his wife. Women’s employment status and their experience of IPV are positively associated with higher prevalence of IPV among employed women. Women in households with male dominance of decision-making had lower prevalence of IPV compared with women in households with no male dominance in decision-making showing negative relationship between male dominance in the family and women’s experience of IPV (β = −0.360; CI: −0.485, −0.234). Partner’s education and partner alcoholic drink were positively associated with women’s experience of IPV, while place of residence was negatively associated with women’s experience of IPV. However, community poverty level and geographical region had mixed relationships with women’s experience of IPV.

**Table 3:** Percentage distribution of ever experience of at least one partner violence by background characteristics and unadjusted binary logistic regression coefficient

Characteristic	% Ever experienced	Coefficient	95% CI
Interparental violence			
Not exposed ^ref^	17.4	–	–
Exposed	44.3	1.327	1.176 1.477
Maternal age			
15–24 years ^ref^	17.9	–	–
25–34 years	21.0	0.198	0.074 0.324
35–49 years	19.2	0.086	−0.063 0.235
Maternal education			
Low ^ref^	17.4	–	–
Moderate	26.0	0.511	0.370 0.652
High	17.1	−0.025	−0.266 0.216
Attitudes towards wife beating			
Violence not justified ^ref^	16.0	–	–
Violence justified	25.7	0.597	0.460 0.734
Employment status			
Not working ^ref^	15.7	–	–
Working	21.3	0.374	0.246 0.501
Male dominance in family			
Not male dominated ^ref^	23.6	–	–
Male dominated	17.7	−0.360	−0.485 −0.234
Partner drinks alcohol			
Does not drink ^ref^	15.5	–	–
Drinks	39.0	1.251	1.109 1.392
Partner’s education			
Low ^ref^	17.1	–	–
Moderate	24.1	0.433	0.296 0.570
High	21.3	0.276	0.076 0.476
Proportion poorest in community			
Low ^ref^	22.0	–	–
Medium	26.1	0.226	−0.068 0.520
High	14.0	−0.553	−0.791 −0.315
Place of residence			
Urban ^ref^	21.3	–	–
Rural	18.6	−0.166	−0.369 0.038
Geographic region			
North-central ^ref^	26.0	–	–
North-east	28.5	0.129	−0.202 0.461
North-west	9.5	−1.205	−1.594 −0.816
South-east	30.0	0.180	−0.163 0.523
South-south	25.6	−0.016	−0.312 0.280
South-west	19.0	−0.404	−0.678 −0.129

Notes: *ref.* reference category

Table 4 presents the fixed effects of the multilevel models. In Model 1 based solely on exposure to interparental violence, women who had exposure to interparental violence were more than five times as likely as non exposed women to experience IPV (OR = 5.356; CI: 3.371–8.509). With the inclusion of selected individual/relationship factors in Model 2, EIPV maintained significant influence on women’s experience of IPV. Women who were exposed to interparental violence were nearly five times as likely as non exposed women to experience IPV (OR = 4.487; CI: 3.047–6.607). In the model, maternal education, attitudes towards wife beating, employment status, and partner alcoholic drink revealed significant effects on the likelihood of experiencing IPV among women. For instance, women who had high educational attainment were 48.4% less likely to experience IPV compared with women who had low educational attainment (OR = 0.516; CI: 0.353–0.754). Likewise, women who think the male partner is justified in beating his wife were nearly twice as likely as women who think otherwise to experience IPV (OR = 1.956; CI: 1.575–2.429), and women whose male partner drink alcohol were six times as likely as women whose male partners do not drink alcohol to experience IPV (OR = 6.043; CI: 3.976–9.185).

**Table 4:** Fixed-effects of multilevel logistic regression

Characteristic	Model 1			Model 2			Model 3		
	Odds ratio	p > |z|	95% CI	Odds ratio	p > |z|	95% CI	Odds ratio	p > |z|	95% CI
Interparental violence									
Not exposed ^ref^	1.000	–	–	1.000	–	–	1.000	–	–
Exposed	5.356	<0.001*	3.371 8.509	4.487	<0.001*	3.047 6.607	4.018	<0.001*	2.626 6.145
Maternal age									
15–24 years ^ref^				1.000	–	–	1.000	–	–
25–34 years				1.154	0.160	0.945 1.410	1.116	0.264	0.920 1.353
35–49 years				0.898	0.328	0.724 1.114	0.866	0.179	0.702 1.068
Maternal education									
Low ^ref^				1.000	–	–	1.000	–	–
Moderate				1.192		0.966 1.470	1.113	0.302	0.908 1.363
High				0.516	0.001**	0.353 0.754	0.493	<0.001*	0.335 0.724
Attitudes toward wife beating									
Violence not justified ^ref^				1.000	–	–	1.000	–	–
Violence justified				1.956	<0.001*	1.575 2.429	1.861	<0.001*	1.484 2.333
Employment status									
Not working ^ref^				1.000	–	–	1.000	–	–
Working				1.416	0.001**	1.163 1.724	1.439	<0.001*	1.179 1.757
Male dominance in the family									
Not male dominated ^ref^				1.000	–	–	1.000	–	–
Male dominated				1.182	0.068	0.988 1.413	1.229	0.022**	1.031 1.466
Partner’s education									
Low ^ref^				1.000	–	–	1.000	–	–
Moderate				1.103	0.335	0.903 1.347	1.028	0.774	0.849 1.246
High				1.040	0.782	0.788 1.372	0.961	0.769	0.736 1.254
Partner drinks alcohol									
Does not drink ^ref^				1.000	–	–	1.000	–	–
Drinks				6.043	<0.001*	3.976 9.185	5.133	<0.001*	3.220 8.183
Proportion poorest in community									
Low ^ref^							1.000	–	–
Medium							1.471	0.134	0.888 2.439
High							0.693	0.158	0.417 1.153
Place of residence									
Urban							1.000	–	–
Rural							0.972	0.873	0.691 1.369
Geographical Region									
North-central ^ref^							1.000	–	–
North-east							3.193	<0.001*	1.720 5.929
North-west							0.079	<0.001*	0.035 0.179
South-east							0.781	0.370	0.454 1.341
South-south							0.713	0.193	0.428 1.187
South-west							0.531	0.018**	0.314 0.896

Notes: *OR* Odds Ratio, *ref.* reference category, *p < 0.01, **p < 0.05

In the full model, women who had exposure to interparental violence were four times as likely as non exposed women to experience IPV (OR = 4.018; CI: 2.626–6.147). In the model, women’s level of education, attitudes towards wife beating, employment status, male dominance in the family, partner alcohol drink, and geographic region were important factors for explaining women’s experience of intimate partner violence. Women who attained high educational level were 50.7% less likely to experience intimate partner violence compared with women who attained low educational level (OR = 0.493; CI: 0.335–0.724). Women who think the male partner is justified in beating his wife were 86.1% more likely to experience intimate partner violence compared with women who did not think the male partner is justified in beating his wife (OR = 1.861; CI: 1.484–2.333). Likewise, employed women were 43.9% more likely to experience intimate partner violence compared with unemployed women (OR = 1.439; CI: 1.179–1.757). Women whose male partner drink alcohol were more than five times likely to experience intimate partner violence compared with women whose male partners do not drink alcohol (OR = 5.133; CI: 3.220; CI: 8.183). Also, women in North-east Nigeria were more likely to experience intimate partner violence compared with women in other parts of the country.

Table 5 presents the random effects of the multilevel models. In the empty model and across the three nested models, the values of the log-likelihood and the AIC reduce consistently to indicate that the three models fitted in the study were a good fit to the data analysed. Based on the empty model, the ICC was 64.4% indicating that in the absence of EIPV as well as the other explanatory variables, there was high variation in women’s experience of intimate partner violence in the population. Though, the ICC in subsequent models reduce consistently, the ICC values however show that community characteristics were also important for explaining variations in women’s experience of intimate partner violence. The ICC values of 63.1% in Model 1, 61.0% in Model 2, and 51.0% in the full model indicate that the contribution of community characteristics to variations in women’s experience of intimate partner violence was high.

**Table 5:** Random effects of multilevel logistic regression

Parameter	Empty model	Model 1	Model 2	Model 3
Community-level variance (S.E.)	5.943 (1.585)	5.633 (1.393)	5.144 (1.107)	3.424 (0.917)
Log likelihood	−9191.156	−9081.895	−8854-555	−8738.863
LR test	χ2 = 3136.6; *p* < 0.001	χ2 = 2716.2; *p* < 0.001	χ2 = 2209.6; *p* < 0.001	χ2 = 1457.87; *p* < 0.001
AIC	18,388.31	18,171.79	17,737.11	17,521.73
ICC (%)	64.4	63.1	61.0	51.0

## Discussion

This study examined the relationship between interparental violence and women’s experience of intimate partner violence in Nigeria based upon data from the 2013 Nigeria Demographic and Health Survey. This communication should prove to be an important contribution to the literature on intimate partner violence and women’s sexual and reproductive health in Nigeria because the issue have not received much attention in previous studies exploring the predictors of intimate partner violence in Nigeria [34–37]. The national representativeness of the data analysed in the current study also enhance generalisation of the findings compared with findings in a previous study [49] conducted among students in tertiary institution in the country. The prevalence of intimate partner violence found in the study was comparable to prevalence found in previous Nigerian studies [34–42].

The study found that EIPV increase women’s risk of intimate partner violence in Nigeria. The study thus gave credence to the possibility of intergenerational transmission of violence in line with the assertion of the theory of intergenerational transmission of violence and consistent with studies across the world that have provided empirical support for the theory [55–60]. The pathway through which violence becomes transmitted from generation to generation is well captured by the social learning theory [51]. Violent acts against spouse may be learnt by children if children witness such aggression. As prescribed by the theory, children observe how parents relate with each other and react to issues within the family. Whenever family violence occurs, children may take note of the consequences experienced by the violated parent and may consider behaving in similar fashion. Where there are no sanctions or punishment for the aggressor, many may grow with the perception that family violence is a normal way of life, and may repeat such behaviour later in life. This is akin to societal attitudes to family violence in many parts of Nigeria. Until recently, violence within marital unions is usually described as a ‘family affair’ even when they are reported to law enforcement agencies. It only becomes an offence, when the violence results in ‘grievous’ hurt such as damage to eyes or nose [63]. This may have encouraged the practice of intimate partner violence, and may as well contribute to continued violation of women’s rights in the country. Increasing evidence of all types of IPV in Nigeria requires expansion of IPV prevention efforts in the country. Existing programmes such as the BCC should be modified to include mechanism for identifying women with both EIPV and IPV experiences. A good understanding of how EIPV shapes the IPV experiences of such women is crucial to programming for altering intergenerational transmission of violence within the social environment. Also, steps could be taken to ensure that attitudes and behaviour pattern formed during childhood as a result of witnessing interparental aggression are moderated through school-based educational programmes. This could be achieved by expanding the curriculum of existing population and family life education to include possible effects of EIPV on future attainable standards of reproductive health.

However, with increasing gender agitation in the country, harmful practices against women have become well recognised in the country with series of efforts (including enactment of laws) being taken to redress the situation. A number of states in the country have enacted laws that prohibit several culturally supported practices that undermine women’s health and rights. The enactment of the Violence against Persons Act of 2015 [66] represents the first all embracing legal framework to reduce violence against persons in the country. Though, the provisions of the law are not women-specific, it however outlaws most forms of violence against women including female genital mutilation, wife battery, forceful ejection of women from households, abandonment of wife and children without means of livelihood, rape, other sexual assaults and harmful widowhood practices. Nevertheless, enforcement of the Act should complement a new social and behaviour change communication (SBCC) programme. This programme should specifically target children who have witnessed interparental violence, and should seek to achieve two things. One, it should seek to change perception that family violence is a ‘family affair’. This may discourage some exposed children from adopting and practicing the type of family violence they have witnessed as a child. Two, the programme should seek to help exposed children to overcome the trauma and challenges of witnessing interparental violence through appropriate counselling and provision of information on the harmful effects of violence on women [15–21], as well as the punishment that awaits perpetrators of intimate partner violence.

Findings from the study further confirmed that both individual/relationship and community characteristics are important for explaining the occurrence and prevalence of partner violence in line with previous studies [25, 27, 29, 30, 36, 50]. In particular, education, employment and attitudes towards wife beating were found to be significantly associated with intimate partner violence. While it was found that the likelihood of partner violence reduces among women with high educational attainment in the country, the likelihood of partner violence increases among employed women. Several public health and social programmes in the country have emphasised expanding women’s access to education as a principal way of boosting women’s social status and reproductive health in the country. However, the impact of education on reducing intimate partner violence may be marginal if improvement in women’s education is not matched by similar improvement in men’s education. The current level of public education in the country should therefore be improved upon by increase funding of educational infrastructure in the country, and where this is not attainable, more family-oriented programmes could be devised to specifically provide education, information and communication about gender-based violence within the family. One likely reason why intimate partner violence was found to be higher among employed women may be conflict arising from how women’s income is spent. In most cases in the country, the male partner wants to have a say in how his spouse earnings is being spent, and whenever this is resisted, intimate partner violence may occur. Such behaviour could however be addressed by more effective community-based behaviour change communication initiatives.

### Limitations

This study suffers from three types of drawbacks. The first is the non inclusion of qualitative data in the analysis. Qualitative data may provide in-depth details of the type of intimate partner violence witnessed by women, its intensity and consequences, as well as the specific circumstances that resulted in intimate partner violence among respondents. These to a great extent may give insight into how violence is transmitted from parents to children, and whether recent intimate partner violence could be linked to specific issue in the past. The study however seeks to provide answer to whether EIPV increase women’s risk of IPV in Nigeria for which the quantitative data analysed was sufficient. The second type of drawback revolves around the data analysed. The data analysed in the study was cross-sectional in nature. This presents a snapshot of parental aggression and intimate partner violence only for the time point of data collection, and may undermine the claim that EIPV cause IPV. However, the findings are sufficient to establish association between EIPV and women’s experience of IPV in Nigeria.

Though, the 2013 NDHS adopted international data collection standard, the possibility of under-reporting of either or both interparental violence and intimate partner violence cannot be ruled out among the respondents, particularly when the interviews were conducted within respondents’ homes. This might encouraged some of the women to give socially desirable responses. Also, under-reporting of intimate partner violence cannot be ruled out among Nigerian women because until recently, intimate partner violence was usually treated as a ‘family affair’ even when they are reported to law enforcement agencies in the country. Finally, interparental violence was captured in the 2013 NDHS by a single question which suggests only physical violence. This may not present a complete picture of family violence witnessed as a child by participants in the survey. One reason that may account for this omission is insufficient attention on the links between exposure to interparental violence and prevalence of intimate partner violence in Nigeria. It is expected that future rounds of the NDHS will develop further questions to capture fully the range of exposures to interparental violence.

The third type of drawback emanates from the method of data analysis. Bivariate and multivariate analyses in the study were based on combination of all types of intimate partner violence. This may limit understanding the associations between specific intimate partner violence and exposure to interparental violence. Though, some previous studies have also separately analysed combined IPV [34, 36], types of IPV have been combined in the study because the proportion of women who experienced at least one type of intimate partner violence was relatively larger, permitting more valid analysis and conclusion regarding association between interparental violence and IPV than are possible with each specific type of intimate partner violence.

## Conclusion

This study investigated the relationship between interparental violence and women’s experience of intimate partner violence in Nigeria by analysing nationally representative data from the 2013 Nigeria Demographic and Health Survey. Findings from the study made contributions to literature on intimate partner violence in Nigeria by providing research evidence that women who witnessed interparental violence have higher likelihood of experiencing intimate partner violence in line with the assertion of the intergenerational transmission of violence theory. The study hypothesis that exposure to interparental violence has significant effect on women’s risk of intimate partner violence was thus verified. The development of more social and behaviour change communication (SBCC) programmes to help children who witnessed interparental aggression overcome the trauma and challenges of the exposure through counselling and education is required in Nigeria.

## Abbreviations

IPV: Intimate partner violence
NDHS: Nigeria Demographic and Health Survey
SBCC: Social and behaviour change communication
WHO: World Health Organization
